# Electrocatalytic Platform Based on Silver-Doped Sugar Apple-like Cupric Oxide Embedded Functionalized Carbon Nanotubes for Nanomolar Detection of Acetaminophen (APAP)

**DOI:** 10.3390/s23010379

**Published:** 2022-12-29

**Authors:** Deepak Balram, Kuang-Yow Lian, Neethu Sebastian

**Affiliations:** 1 Department of Electrical Engineering, National Taipei University of Technology, No. 1, Section 3, Zhongxiao East Road, Taipei 106, Taiwan; 2 Institute of Organic and Polymeric Materials, Department of Molecular Science and Engineering, National Taipei University of Technology, No. 1, Section 3, Zhongxiao East Road, Taipei 106, Taiwan

**Keywords:** electrochemical sensor, acetaminophen, analgesic drug, ternary nanocomposite, carbon nanotubes, cupric oxide

## Abstract

Economical and nanomolar-level determination of the analgesic drug, acetaminophen (APAP), is reported in this work. A novel ternary nanocomposite based on silver-doped sugar apple-like cupric oxide (CuO)-decorated amine-functionalized multi-walled carbon nanotubes (fCNTs) was sonochemically prepared. CuO nanoparticles were synthesized based on the ascorbic acid-mediated low-temperature method, and sidewall functionalization of CNTs was carried out. Important characterizations of the synthesized materials were analyzed using SEM, TEM, HAADF-STEM, elemental mapping, EDX, lattice fringes, SAED pattern, XRD, EIS, UV-Vis, micro-Raman spectroscopy, and FTIR. It was noted the sonochemically prepared nanocomposite diligently fabricated on screen-printed carbon electrode showcased outstanding electrocatalytic performance towards APAP determination. The APAP sensor exhibited ultra-low limit of detection of 4 nM, wide linear concentration ranges of 0.02–3.77 and 3.77–90.02 μM, and high sensitivity of 30.45 μA μM^−1^ cm^−2^. Moreover, further evaluation of the sensor’s performance based on electrochemical experiments showcased outstanding selectivity, stability, reproducibility, and repeatability. Further, excellent practical feasibility of the proposed APAP sensor was affirmed with excellent recovery larger than 96.86% and a maximum RSD of 3.67%.

## 1. Introduction

Acetaminophen (N-acetyl-p-aminophenol) (APAP) is a well-known non-narcotic analgesic and antipyretic drug used worldwide as a mild-to-moderate pain reliever and is a common medication to reduce fever. Apart from fever and pain relief, APAP is widely used as an over-the-counter drug for common illnesses, such as cold, osteoarthritis, allergy, and headache. Moreover, APAP monotherapy is recommended to reduce fever in patients with COVID-19 as a targeted antiviral treatment for COVID-19 is currently not available [1,2,3]. In this scenario, the development of an APAP sensor is considered highly significant. Even though APAP is used widely for the aforementioned illnesses, it is to be noted its over dosage leads to hepatotoxicity [4,5]. The APAP toxicity is critical as it causes acute liver failure [6], pancreatitis [7], renal impairment [8], lactic acidosis [9], kidney cancer [10], and skin rashes [11] in human beings. Studies show APAP is a main reason for liver damage in the United States and United Kingdom, and in the United States alone, around 100,000 cases of APAP toxicity are reported every year [12]. A single dose of APAP above 10 g or multiple smaller doses in a day in adults may lead to toxicity. Moreover, investigations show in some people, even normal doses of APAP can lead to toxicity, and it is worth mentioning toxic doses of APAP vary among people [13]. Therefore, the accurate detection of APAP in biological samples is crucial and is a necessity in pharmaceutical formulations for quality control of medicine.

Even though various methods, such as titrimetry, spectrophotometry, high-performance liquid chromatography (HPLC), and chemiluminescence have been applied for APAP detection, these methods suffer some shortcomings. For instance, the titrimetric, spectrophotometric, and chemiluminescence methods involve tedious extraction processes prior to detection. Liquid chromatography is time consuming, making it unsuitable for rapid APAP determination. On the other hand, electrochemical detection is a powerful analytical technique with advantages of instrumental simplicity, low cost, high sensitivity, and portability [14,15,16]. Since most electroanalytic techniques are selective and capable of highly sensitive and rapid measurements over a wide linear range, require no sample preparation, and given the fact that APAP is electroactive, the electrochemical technique is adopted in this work for its determination.

As modifying the electrode surface enhances electrode kinetics, thereby improving selectivity and sensitivity of electrochemical sensors, the preparation of a modified electrode using materials with excellent electrocatalytic activity is significant [17]. Transition metal oxide nanoparticles as a propitious material has significant importance in the field of electrochemistry [18,19,20]. In this regard, cupric oxide (CuO)-based nanomaterials attained much recognition due to distinctive physiochemical properties. Apart from its exceptional properties, some of the significant features of CuO worth mentioning here are its non-toxicity, cost-effectiveness, and earth-abundance. As engineering the morphology of materials reinforces its properties, different morphologies of CuO, including nanowires, hexapods, nanospheres, nanosheets, nanocubes, and nanoflakes have been synthesized [21,22,23,24,25,26]. The high specific surface area along with excellent electron transfer and mechanical strength make CuO an ideal material for electrochemical sensor development. Moreover, the high chemical stability of CuO and its ability to promote the electron transfer reaction at low overpotential are significant in catalysis. Therefore, because of the aforementioned unique properties and features of CuO, including its excellent catalytic ability, it was synthesized for the development of an economical and sensitive APAP electrochemical sensor. Silver with the highest electrical conductivity among metals possess various outstanding properties suitable for electrochemical sensor development, such as a large surface-to-volume ratio, facilitates electron transfer, non-toxicity, and biocompatibility [27]. Moreover, various research have reported the excellent electrocatalytic activity of silver nanoparticles [28,29,30]. Hence, CuO was doped with Ag nanoparticles in this work.

Hybrid nanocomposites based on carbon nanotubes (CNTs) are an emerging class of materials that attained great interest among researchers recently [31,32]. By combining CNTs with suitable metal oxide nanoparticles, hybrid nanocomposite with superior physical and chemical properties could be attained [33,34]. Moreover, the side walls of multi-walled carbon nanotubes (MWCNTs) are appropriate for the decoration of metal nanoparticles in the preparation of hybrid nanocomposites. Researchers reported superior performance of hybrid nanocomposites based on CNTs and metal oxide nanoparticles in electrochemical applications [35]. The fact that morphology and size of nanomaterials have significant impacts on various properties of a material, including the electrocatalytic property, signifies the importance of advanced synthetic approaches. Various approaches were adopted in the preparation of hybrid nanocomposites, among which the sonochemical method has significant merits. Excellent crystallinity, enhanced specific surface area, porosity, and improved rate of chemical reactions are some of the advantages of the sonochemical approach, which has a direct correlation to electrochemical performance. Moreover, the sonochemical approach is comparatively simple and inexpensive, and therefore, this non-hazardous acoustic method is suitable for the preparation of hybrid nanocomposites for low-cost sensors [36]. Hence, in this work, Ag-doped CuO-decorated fCNTs were sonochemically prepared for nanomolar level detection of APAP.

To the best of our knowledge, the detection limit of 4 nM for the proposed APAP sensor is one of the lowest. Moreover, there were no previous works on APAP detection using silver-doped cupric oxide, which is an apt material for the development of economical sensors. Electrochemical sensors based on low-cost materials that provide high performance detection are significant as these types of sensors can be commercialized effectively. Furthermore, this work was carried out on screen-printed carbon electrode (SPCE) instead of glassy carbon electrode (GCE), which signifies more practicality to commercialize this sensor.

## 2. Materials and Methods

### 2.1. Materials and Instrumentation

Chemicals, including ascorbic acid (C_6_H_8_O_6_, 99%), sodium nitrate (NaNO_2_), APAP (C_8_H_9_NO_2_), disodium phosphate (Na_2_HPO_4_), monosodium phosphate (NaH_2_PO_4_), ethylenediamine (EDA), copper sulfate pentahydrate (CuSO_4_.5H_2_O), and CNTs are from Sigma-Aldrich. Metrohm Autolab was utilized for conducting electrochemical experiments, and 0.05 M phosphate buffer solution (PBS) was used as the supporting electrolyte in all these experiments. Hitachi S4800 instrument was utilized to perform scanning electron microscopy (SEM) analysis. Transmission electron microscopy (TEM), high resolution transmission electron microscopy (HR-TEM), and elemental mapping were carried out using JEOL JEM2100F instrument. X-ray diffraction (XRD) was conducted with Bruker D8 advanced diffractometer utilizing Cu-Kα radiation. JASCO V770 was utilized for analyzing absorbance spectra of materials, Raman spectrum was captured with Acron confocal system, and Fourier transform infrared (FTIR) analysis was conducted with PerkinElmer spotlight 200i spectrometer.

### 2.2. Ascorbic Acid-Mediated Synthesis of Ag-Doped CuO Nanoparticles

CuO nanoparticles were prepared based on the ascorbic acid-modified synthesis method. Initially, 0.03 M CuSO_4_.5H_2_O was dissolved in 100 mL deionized (DI) water. Thenceforth, 40 mL 0.1 M NaOH was added to the prepared solution. Then, the mixture was magnetically stirred for 30 min. After thorough stirring of the mixture, 20 mL 0.1 M ascorbic acid was added. This was followed by 2 h of the magnetic stirring process. Afterwards, the obtained product was washed and centrifuged. Later, the product was dried at 60 °C overnight, and in the final stage, calcination was performed at 450 °C, 1 h for obtaining CuO. Afterwards, 1 g of CuO was dispersed in 10 mL water, and a certain quantity of AgNO_3_ was weighed. Then AgNO_3_ was transferred into the CuO dispersion under stirring. The mixture was magnetically stirred in the dark for 1 h. After continuous stirring in the dark, the mixture was taken for the irradiation of UV light for 30 min. Finally, it was centrifuged, washed, and dried overnight.

### 2.3. Synthesis of fCNTs

In the preparation of fCNTs, primarily, 0.5 g pristine CNTs was treated with 200 mL H_2_SO_4_/HCl (3:1) under reflux for 16 h. Then, it was washed with DI water until pH 7 was attained. Afterwards, it was dried at 50 °C overnight. Then, 300 mg purified CNTs attained from the above steps and 300 mg NaNO_2_ were dispersed in 30 mL EDA solution and then ultrasonicated for 1 h. Eventually, the suspension was moved to a water bath at 80 °C for 2 h, and then, the product was centrifuged, washed, and dried at 50 °C overnight.

### 2.4. Preparation of AgCuO NPs/fCNTs Modified Electrode

**Scheme 1:**
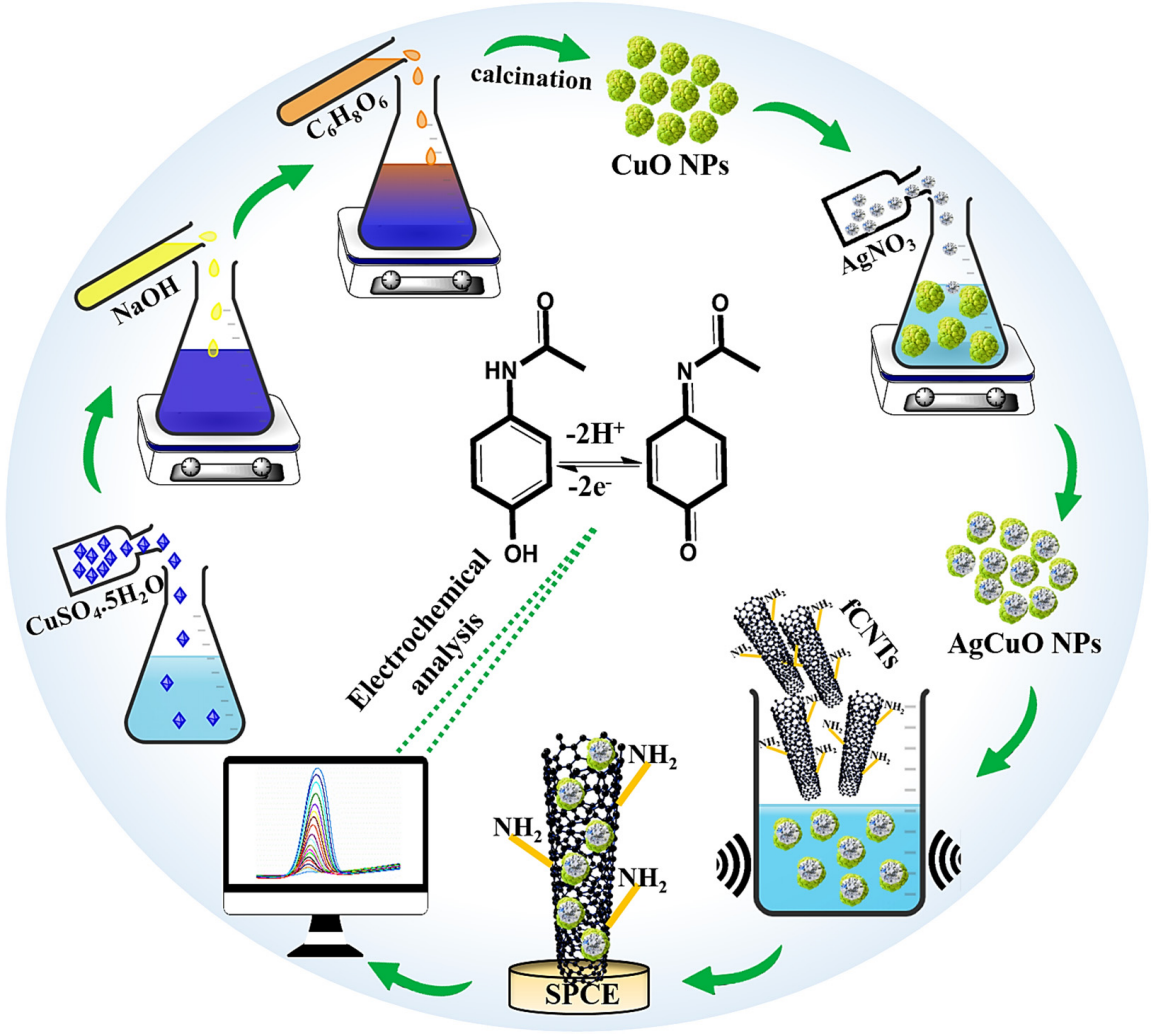
Illustration of AgCuO NPs/fCNTs preparation for APAP detection.

In the preparation process of AgCuO NPs/fCNTs, 75 mg of the synthesized AgCuO NPs was taken and mixed with DI water. Then, sonication of CuO solution for 1 h was performed. Afterwards, 25 mg of synthesized fCNTs was added to the ultrasonicated solution. After this, the AgCuO NPs/fCNTs mixture was magnetically stirred and again ultrasonicated for 2.5 h to procure AgCuO NPs/fCNTs. As part of the electrode fabrication, primarily, sonochemically prepared nanocomposite was dispersed in ethanol. Thenceforth, drop casting 8 µL AgCuO NPs/fCNTs solution on working electrode (SPCE) was laboriously carried out and was dried at 60 °C. The extra-bound AgCuO NPs/fCNTs that was not correctly attached on the SPCE surface was removed by meticulously washing the SPCE surface with DD water. Then, this fabricated AgCuO NPs/fCNTs/SPCE was used in this work for various electrochemical investigations. Scheme 1 shows the preparation of AgCuO NPs/fCNTs nanocomposite for the detection of APAP.

## 3. Results and Discussions

### 3.1. Characterization of Synthesized Materials

#### 3.1.1. XRD Analysis

**Figure 1:**
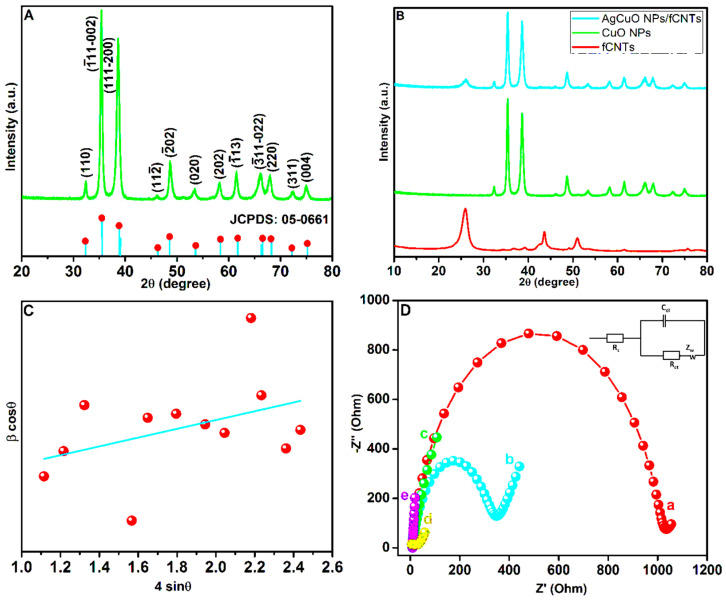
XRD: (**A**) CuO; (**B**) AgCuO NPs/fCNTs, CuO, and fCNTs. (**C**) Williamson–Hall plot of CuO. (**D**) Nyquist plot of bare SPCE (a), CuO/SPCE (b), fCNTs/SPCE (c), AgCuO NPs/SPCE (d), and AgCuO NPs/fCNTs/SPCE (e).

Foremost, XRD patterns of prepared materials were recorded. The XRD peaks of synthesized CuO is shown in Figure 1A with diffraction peaks 32.37°, 35.40°, 38.62°, 46.14°, 48.71°, 53.33°, 58.18°, 61.47°, 66.09°, 67.91°, 72.34°, and 75° representing diffraction planes (110), ($\bar{1}11$–002), (111–200), ($11\bar{2}$), ($\bar{2}02$), (020), (202), ($\bar{1}13$), ($\bar{3}11$), (220), (311), and (004), respectively.

The XRD patterns of fCNTs given in Figure 1B shows characteristic peak 25.93° corresponding to plane (002), and peaks representing planes (100) and (004) was observed at 43.67° and 51.04°, respectively. The XRD patterns of AgCuO NPs/fCNTs are shown in Figure 1B. All diffraction peaks of CuO and characteristic intense peak of fCNTs representing plane (002) are present in the pattern without much shift in their degrees. This proves the successful preparation of AgCuO NPs/fCNTs.

The crystallite size of CuO calculated based on Debye–Scherrer equation [37] is 17.26 nm, and using the Williamson–Hall method [38], the micro strain for CuO nanoparticles obtained is 0.002. The Williamson–Hall plot of CuO nanoparticles is given in Figure 1C.

#### 3.1.2. Analysis of Electrochemical Impedance Spectroscopy (EIS) Spectra

EIS profiles of bare SPCE, CuO NPs/SPCE, AgCuO NPs/SPCE, fCNTs/SPCE, and AgCuO NPs/fCNTs/SPCE were recorded and shown in Figure 1D. The comparatively best electrical conductivity was observed for AgCuO NPs/fCNTs/SPCE. The charge transfer resistance (R_CT_) of bare SPCE, CuO NPs/SPCE, fCNTs/SPCE, AgCuO NPs/SPCE, and AgCuO NPs/fCNTs/SPCE are 1035.6, 349.7, 9.04, 27.44, and 8.89 Ω, respectively. Thus, the results suggest good electrical conductivity in AgCuO NPs/fCNTs/SPCE, which can provide excellent electron transfer between the electrode and electrolyte interfaces.

#### 3.1.3. Morphology and Elemental Composition

**Figure 2:**
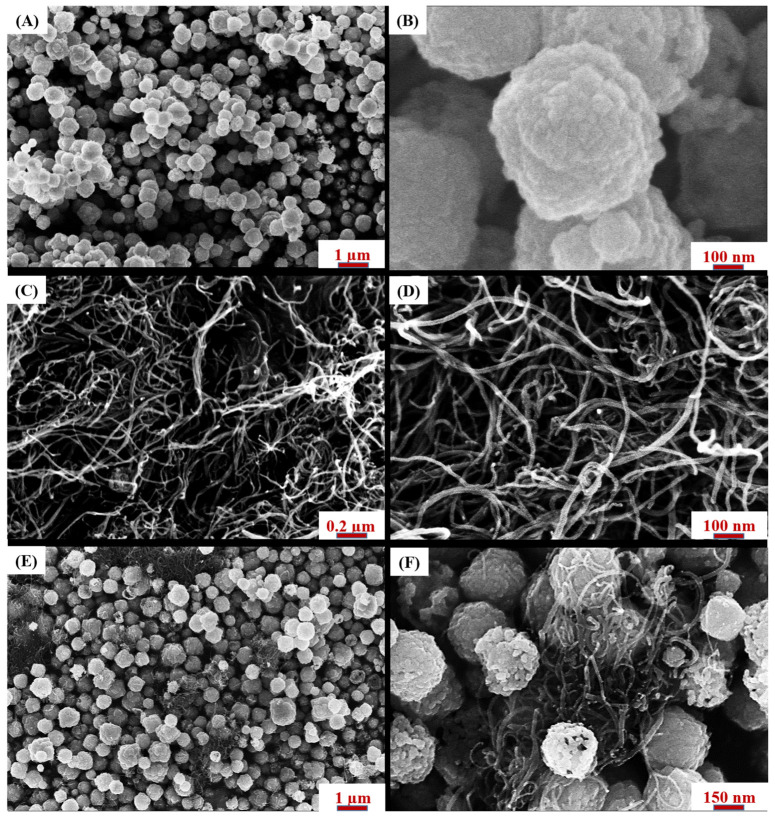
SEM: (**A**,**B**) CuO nanoparticles; (**C**,**D**) fCNTs; (**E**,**F**) AgCuO NPs/fCNTs.

**Figure 3:**
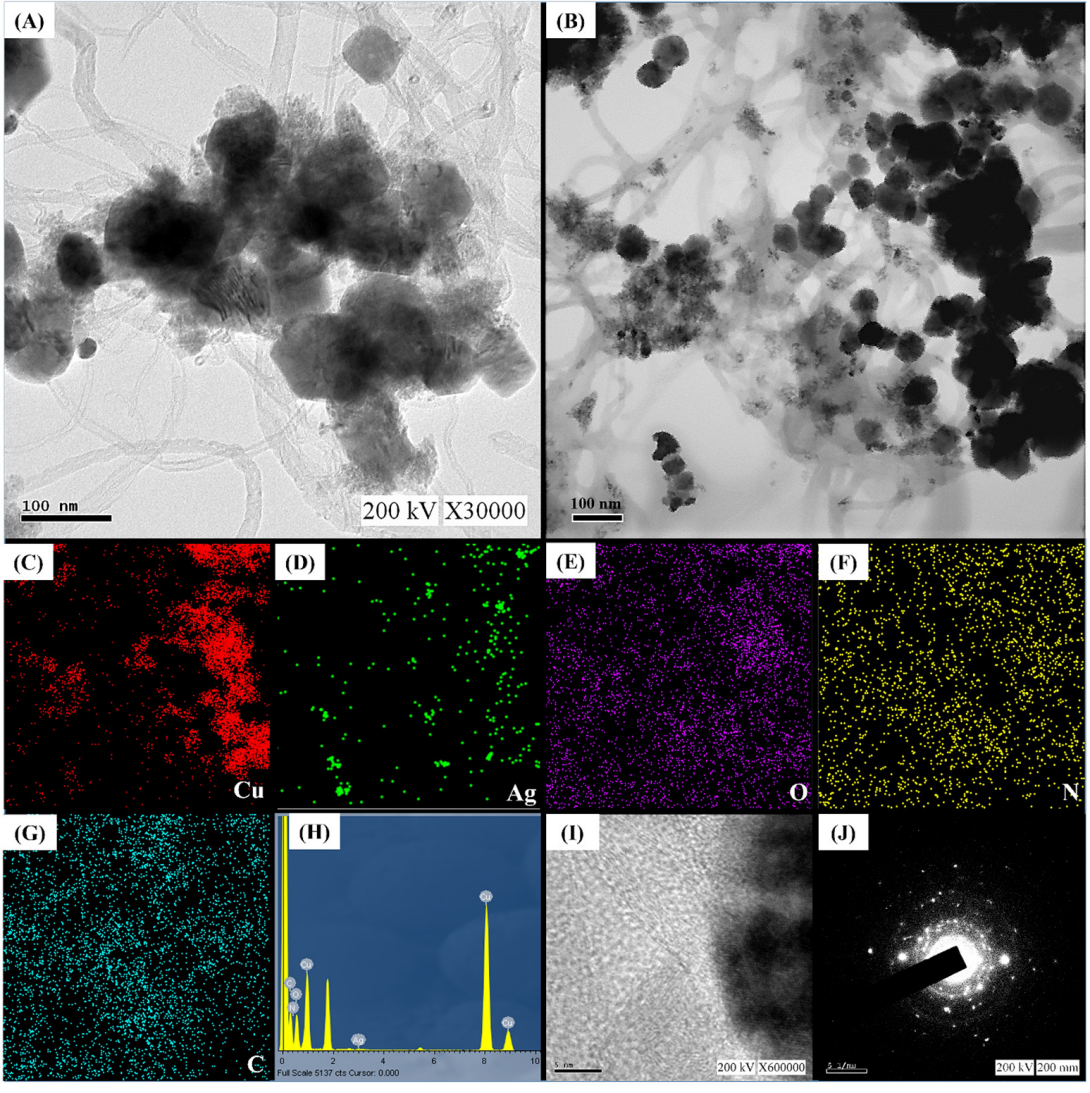
(**A**) HR-TEM, (**B**) HAADF-STEM, (**C**–**G**) corresponding elemental mapping of copper, silver, oxygen, nitrogen, and carbon, (**H**) EDX, (**I**) lattice fringes, and (**J**) SAED patterns of AgCuO NPs/fCNTs.

SEM of CuO, fCNTs, and AgCuO NPs/fCNTs was recorded as part of this study. When we analyze the microscopy image of CuO in Figure 2A,B, morphology similar to sugar apple fruit can be observed. The synthesized CuO has thick rind composed of a knobby, segment-like structure as in sugar apple. The diameter of the single CuO nanoparticle is around 600 nm. The SEM of fCNTs is captured and shown in Figure 2C,D. This SEM image shows the typical tube-like morphology of fCNTs. The mean diameter of a single tube of fCNT was measured and is around 20–25 nm. Figure 2E,F show the SEM image of AgCuO NPs/fCNTs. The presence of CuO and fCNTs arranged randomly can be noticed in the SEM of nanocomposite. The HR-TEM of the nanocomposite in Figure 3A shows the CuO nanoparticles and fCNTs were well wrapped together. The high angle annular dark-field imaging–scanning transmission electron microscopy (HAADF-STEM) (Figure 3B) of AgCuO NPs/fCNTs and its respective energy dispersive X-ray (EDX) spectra (Figure 3H) and elemental mapping (Figure 3C–G) proves the presence of Cu, Ag, O, N, and C without impurities. Lattice fringes shown in Figure 3I have an interplanar spacing of 0.2951 nm, and the selected area electron diffraction (SAED) pattern shown in Figure 3J proves the nanocrystalline nature of the nanocomposite.

#### 3.1.4. Analysis of Raman Spectra

**Figure 4:**
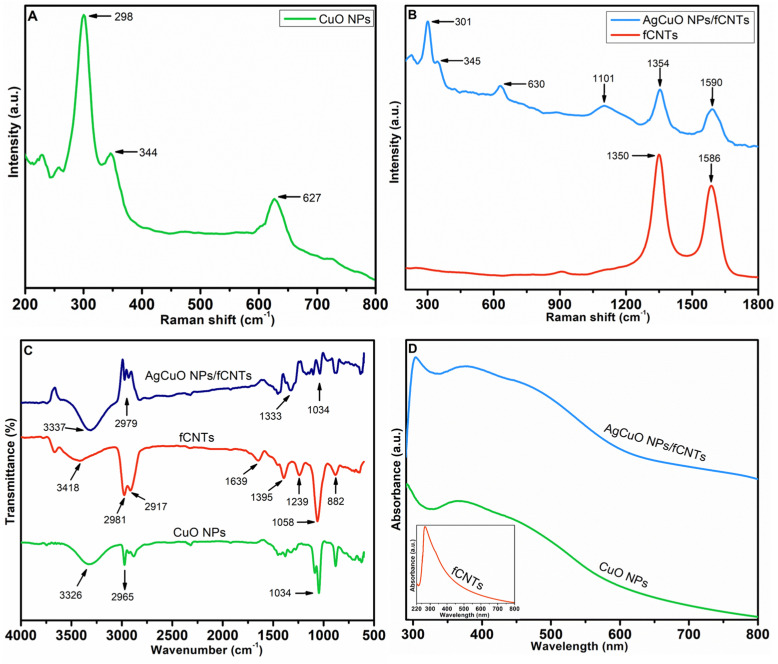
Raman spectrum: (**A**) CuO; (**B**) fCNTs and AgCuO NPs/fCNTs. (**C**) FTIR spectrum: CuO, fCNTs, and AgCuO NPs/fCNTs. (**D**) Absorbance spectrum of CuO and AgCuO NPs/fCNTs. Inset shows spectra of fCNTs.

Advanced micro-Raman spectroscopy of CuO nanoparticles, fCNTs, and AgCuO NPs/fCNTs were recorded and are shown in Figure 4. Raman spectroscopy of CuO in Figure 4A shows well-defined peaks at certain wavelengths. CuO has a space group symmetry $C_{2h}^{6}$ having 12 optical-phonon modes. Among these, six are infrared active modes (3A_u_ + 3B_u_), three are acoustic modes (1A_u_ + 2B_u_), and the remaining three are Raman active modes (1A_g_ + 2B_g_). The strongest peak of CuO spectra at 298 cm^−1^ represents A_g_ mode, and peaks 344 and 627 cm^−1^ denote B_g_ mode. The presence of all three Raman active modes in CuO nanoparticles confirms the existence of the Cu-O bond [39]. Micro-Raman spectra of fCNTs was recorded in this study and is shown in Figure 4B. The peaks 1350 and 1586 cm^−1^ denote the D and G bands, respectively. Spectra of composite was recorded as in Figure 4B, and it shows peaks at 301, 345, and 630 cm^−1^ denoting A_g_ and B_g_ modes of CuO nanoparticles. Moreover, peak 1101 cm^−1^ represents multiphonon band of CuO nanoparticles in AgCuO NPs/fCNTs, and D and G bands can be found at 1354 and 1590 cm^−1^, respectively.

Disorder degree was estimated by calculating I_D_/I_G_ of fCNTs and AgCuO NPs/fCNTs using Raman spectroscopy data. The I_D_/I_G_ ratio is 1.22 for fCNTs and 1.09 for AgCuO NPs/fCNTs. Hence, AgCuO NPs/fCNTs having lower I_D_/I_G_ has few disorders than fCNTs revealing its good structural quality.

#### 3.1.5. Analysis of FTIR Spectra

Analyzing FTIR spectra of CuO nanoparticles shown in Figure 4C, a stronger band at wavenumber 3326 cm^−1^ corresponding to asymmetric O-H stretching vibrations of surface hydroxyl groups of adsorbed water molecules can be observed. The peak 2965 cm^−1^ represents symmetric O-H stretching in CuO nanoparticles, and peak at 1034 cm^−1^ represents Cu-(OH) stretching. Moreover, small peaks at further lower wavenumbers may be a result of Cu-O vibrations. The FTIR spectrum of fCNTs shown in Figure 4C shows scissoring in-plane N-H bending vibrations in amine group denoted by 1639 cm^−1^. The C-N stretching vibration in fCNTs corresponds to peaks 1395 and 1239 cm^−1^, and C=C stretching in fCNTs are denoted by intense peak at 1058 cm^−1^. Apart from inplane N-H vibrations, fCNTs also possess the out-plane N-H bending vibrations and is represented by peak 882 cm^−1^. Two neighboring peaks are observed at 2981 and 2917 cm^−1^ in FTIR of fCNTs corresponding to C-H stretching in fCNTs. Finally, 3418 cm^−1^ represents -NH stretching in fCNTs [40]. The FTIR spectra of AgCuO NPs/fCNTs was also taken and shown in Figure 4C. This spectrum shows well-defined peak at 1034 cm^−1^ similar to the peak observed in CuO. This indeed substantiates that CuO nanoparticles are rightly present in AgCuO NPs/fCNTs. Moreover, peak 1333 cm^−1^ denote C-N stretching in fCNTs. The peak broadened and shifted in the nanocomposite because of overlapping with the CuO bands observed at the same wavenumber. The peak around 2979 cm^−1^ is a result of symmetric O-H vibrations in CuO and C-H stretching vibrations in fCNTs. Peak 3337 cm^−1^ denotes overlapping of O-H vibration in surface hydroxyl groups present in CuO nanoparticles and -NH stretching in fCNTs.

#### 3.1.6. Analysis of Absorbance Spectra

The ultraviolet-visible (UV-Vis) spectra of CuO nanoparticles, fCNTs, and AgCuO NPs/fCNTs are shown in Figure 4D. The UV-Vis spectrum of CuO nanoparticles in Figure 4D shows absorbance peak at wavelength 364 nm, which represents the characteristic band of CuO [41]. An intense absorbance peak at 269 nm can be observed in the spectrum of fCNTs in inset of Figure 4D. This peak indeed represents the π-π* transition in fCNTs. The absorbance spectra of AgCuO NPs/fCNTs shown in Figure 4D indicates absorbance peaks at 293 and 364 nm. The peak at 293nm represents π-π* transition of fCNTs, and the peak at 364 nm is very similar to the characteristic peak observed in CuO nanoparticles. This observation substantiates the fact there was good interaction of fCNTs and CuO nanoparticles in AgCuO NPs/fCNTs formation.

### 3.2. Electrochemical Analysis of APAP

**Figure 5:**
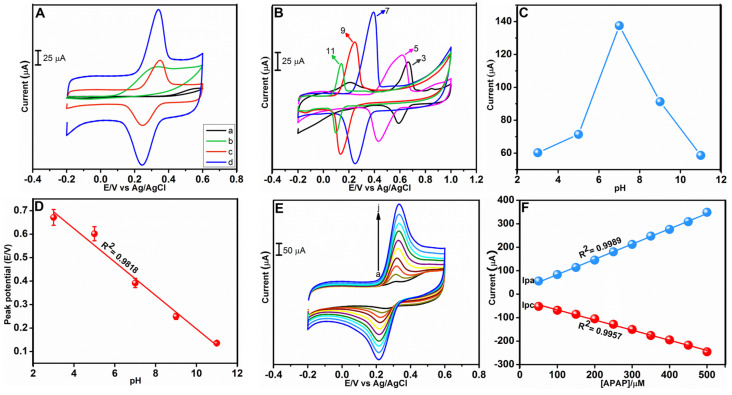
(**A**) CV curves of unmodified SPCE (a), AgCuO NPs/SPCE (b), fCNTs/SPCE (c), and AgCuO NPs/fCNTs/SPCE (d) in the presence of 200 μM APAP at 0.1 V/s. (**B**) CV curves of AgCuO NPs/fCNTs/SPCE at different pH. (**C**) pH vs. oxidation peak current. (**D**) pH Vs. oxidation peak potential. (**E**) CV curves of AgCuO NPs/fCNTs/SPCE during continuous APAP addition from 50 μM to 500 μM (a–j). (**F**) Anodic peak currents (*I*_pa_) and cathodic peak currents (*I*_pc_) against APAP concentration.

**Scheme 2:**
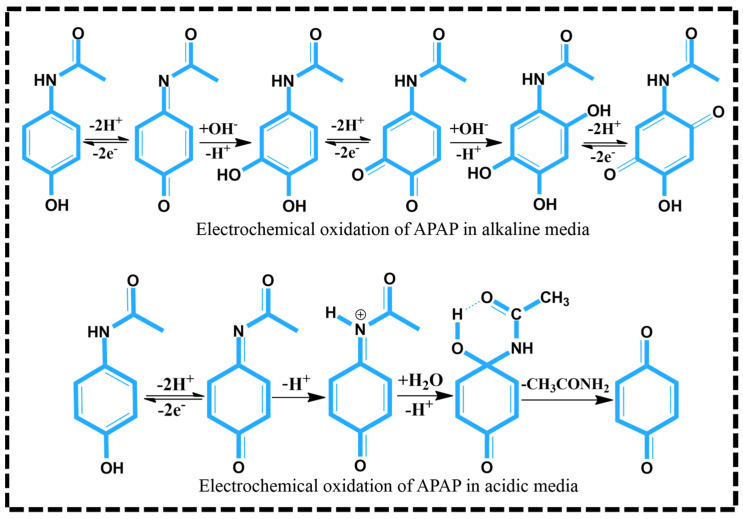
Electrochemical oxidation of APAP.

The cyclic voltammetry (CV) technique was used to analyze and compare the performance of modified electrodes, including AgCuO NPs/SPCE, fCNTs/SPCE, AgCuO NPs/fCNTs/SPCE, and bare SPCE. Figure 5A shows voltametric responses for all modified electrodes considered in this experiment. This comparative evaluation experiment was carried out in existence of 200 μM APAP at 0.1 V/s. Examining Figure 5A, the comparatively weakest voltametric response was attained for bare SPCE, indicating its inferior electrocatalytic activity. Evaluating voltametric response of fCNTs/SPCE in Figure 5A, redox peaks at 0.35 and 0.24 V can be noticed. The voltametric response of AgCuO NPs/SPCE resulted in anodic peak having peak potential of 0.34 V, and AgCuO NPs/fCNTs/SPCE resulted in redox peaks at 0.33 and 0.24 V. Thus, AgCuO NPs/fCNTs/SPCE has resulted in redox peak at comparatively the lowest peak potential. It was AgCuO NPs/fCNTs/SPCE that showed a comparatively better voltametric response towards APAP detection with highly intense oxidation peak current (10 times bare SPCE) corresponding to oxidation of APAP to N-acetyl-p-benzoquinoneimine. The extremely reactive and less stable N-acetyl-p-benzoquinoneimine undergoes dimerization in intermediate pH [42], whereas the high reactivity and unstable nature of N-acetyl-p-benzoquinoneimine in alkaline and acidic media lead to hydroxylation and hydrolysis, respectively, to rapidly form the final product as in Scheme 2.

#### 3.2.1. Impact of pH

This experiment was performed using cyclic voltammetry by placing AgCuO NPs/fCNTs/SPCE in 0.05 M PBS containing 200 μM APAP. Figure 5B shows the voltametric response obtained by carrying out this experiment in various pH of electrolyte. These curves show the redox peak potential decreases with increase in pH, and lowest potential 0.13 V was achieved at pH 11 and highest 0.67 V at pH 3. Redox peak currents of voltametric curve increases from pH 3 to pH 7 and decreases beyond pH 7. Hence, it is understood the strongest redox peak currents can be obtained when the analyte pH is maintained at 7. From the CV curves obtained from this experiment, calibration plots of pH against peak current and potential were shown in Figure 5C,D, respectively, and the regression equation is *E*_pa_ (V) = −0.0712pH + 0.9082 (R^2^ = 0.9818) with slope −71.2 mV/pH. This slope value deviates from the theoretical slope −59 mV/pH as per Nernst equation specifying acetaminophen oxidation process at AgCuO NPs/fCNTs/SPCE is complex.

#### 3.2.2. Effect of Concentration, Scan Rate, Electroactive Surface Area

Effect of concentration was analyzed using cyclic voltammetry by consecutively adding APAP (50–500 μM) to 0.05 M PBS at 0.1 V/s, and the results are presented in Figure 5E. Analyzing these voltametric responses, it is apparent the peak current rises linearly with APAP concentration. It is noted this increase in redox peak currents is at almost the same rate for different concentrations as shown in Figure 5F, and the regression equations calculated from this plot are *I*_pa_ = 0.6498x + 18.552 (R^2^ = 0.9989) and *I*_pc_ = −0.4319x − 23.6686 (R^2^ = 0.9957). From this steady increase of peak currents with concentration of APAP, it could be inferred that AgCuO NPs/fCNTs/SPCE is suitable for electrochemical detection of APAP.

**Figure 6:**
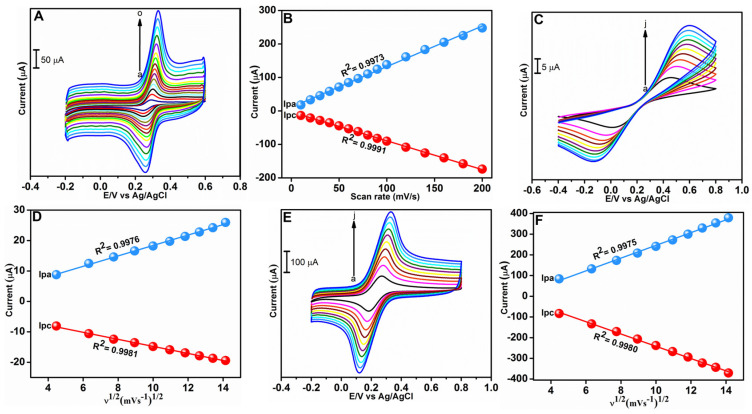
(**A**) CV curves of AgCuO NPs/fCNTs/SPCE at different scan rates from 0.01–0.2 V/s (a–o). (**B**) Anodic (*I*_pa_) and cathodic (*I*_pc_) peak currents against scan rate. Voltametric response in ferricyanide system for various scan rates from 0.02–0.2 V/s (a–j) at (**C**) bare SPCE and (**E**) AgCuO NPs/fCNTs/SPCE. Redox peak current vs. square root of scan rate at (**D**) bare SPCE and (**F**) AgCuO NPs/fCNTs/SPCE.

The scan rate impact in APAP determination was evaluated by recording CV curves of AgCuO NPs/fCNTs/SPCE under different scan rates (0.01 V/s–0.2 V/s), and the voltametric responses are given in Figure 6A. From these voltametric responses, linearity of redox peak current against scan rate was analyzed, and the calibration plot is given in Figure 6B. The corresponding regression equations are *I*_pa_ = 1.2126x + 11.160 (μA, mV s^−1^, R^2^ = 0.9973) and *I*_pc_ = −0.860x − 3.034 (μA, mV s^−1^, R^2^ = 0.9991), respectively. Furthermore, log (*I*_pa_, *I*_pc_) vs. log (scan rate) was evaluated and affirmed electrochemical mechanism at AgCuO NPs/fCNTs/SPCE is adsorption directed [43].

The electroactive surface area of AgCuO NPs/fCNTs/SPCE is analyzed in this work as it is an important factor that influences the electrocatalytic activity. The voltametric curves of bare SPCE in ferricyanide system and its linear plot are provided in Figure 6C,D, respectively, whereas, the respective voltametric curves of AgCuO NPs/fCNTs/SPCE and its linear plot are provided in Figure 6E,F, respectively. The determined electroactive surface area of bare SPCE and AgCuO NPs/fCNTs/SPCE are 0.01 cm^2^ and 0.26 cm^2^, respectively. An in-depth detailing of the electroactive surface area analysis is provided in the Supplementary Information.

#### 3.2.3. Electrochemical Determination of APAP

**Figure 7:**
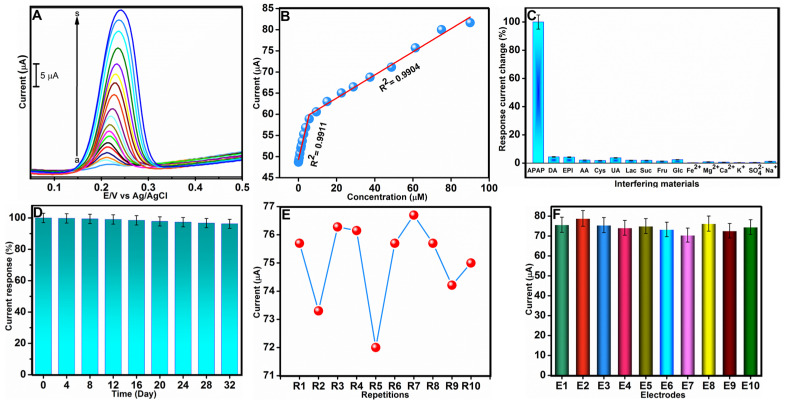
(**A**) DPV curves at AgCuO NPs/fCNTs/SPCE under different APAP concentrations (a–s). (**B**) Peak currents vs. APAP concentrations. (**C**) Selectivity. (**D**) Stability. (**E**) Repeatability. (**F**) Reproducibility.

Differential pulse voltammograms of AgCuO NPs/fCNTs/SPCE under various APAP concentrations were captured, and the results are shown in Figure 7A. Analysis of differential pulse voltammetry (DPV) curves in Figure 7A and the corresponding linear plot in Figure 7B show a steady rise in oxidation peak currents with APAP concentrations. The regression equation determined from this linear plot for the lower concentration range of 0.02–3.77 µM is I (µA) = 2.2268x + 48.94 (R^2^ = 0.9911), and for the higher concentration range of 3.77–90.02 µM it is I (µA) = 0.2735x + 58.358 (R^2^ = 0.9904). An ultra-low-detection limit 0.004 µM for APAP detection was achieved in this DPV analysis. In addition, excellent sensitivity of 30.45 µAµM^−1^cm^−2^ was exhibited by AgCuO NPs/fCNTs/SPCE toward APAP detection. From this DPV analysis which resulted in exceptional sensitivity, LOD, and linear range, it could be affirmed AgCuO NPs/fCNTs/SPCE is excellent in APAP detection. The performance of AgCuO NPs/fCNTs/SPCE was compared with different electrodes toward APAP detection, and the details are shown in Table 1.

#### 3.2.4. Sensor Performance Evaluation Based on Selectivity, Reproducibility, Stability, and Repeatability

In this work, 10-fold excess concentrations of dopamine (DA), epinephrine (EPI), ascorbic acid (AA), L-cysteine (Cys), uric acid (UA), lactose (Lac), sucrose (Suc), fructose (Fru), and glucose (Glc) and 300-fold ferrous (Fe^2+^), magnesium (Mg^2+^), calcium (Ca^2+^), potassium (K^+^), sulfate ($SO_{4}^{2-}$), and sodium (Na^+^) were used for selectivity analysis. The variations in response current from this anti-interference experiment are shown in Figure 7C. Examining this bar diagram, it is apparent the just negligible variation occurs in APAP peak current response. The maximum error in response current observed is only 4.4% (in the presence of DA), which is inconsequential. From these experimental results, the outstanding anti-interference property of APAP sensor could be confirmed.

Experiment was conducted for a period of 32 days to investigate the stability of the APAP sensor. Figure 7D shows a bar diagram showing the peak current response every four days. Examining Figure 7D, only negligible peak current response variation was observed at the end of 32 consecutive days. When we calculated peak current response variation of the proposed APAP sensor after 32 days with respect to that of the first day, we found it is only 3.72%. This negligible variation in peak current response after 32 days indicates good stability of the APAP sensor.

An experiment was performed for analyzing repeatability of APAP sensor. In this experiment, voltametric responses of the proposed APAP sensor were recorded 10 times, and the peak currents are noted. Figure 7E shows the peak current values of the proposed sensor in APAP detection during 10 successive repetitions. The relative standard deviation (RSD) of peak currents was evaluated and is merely 1.97%. From the aforementioned results, it is apparent the APAP sensor using AgCuO NPs/fCNTs exhibits an exceptional repeatability property.

For reproducibility analysis, 10 identical AgCuO NPs/fCNTs/SPCEs were needed, and hence, these 10 electrodes were fabricated separately in the initial stage of this experiment. In the next stage, voltametric responses of 10 identical AgCuO NPs/fCNTs/SPCEs in existence of APAP were recorded. Figure 7F shows a bar diagram that showcases peak current responses for each of the 10 identical AgCuO NPs/fCNTs/SPCEs. Analyzing the Figure 7F, it is evident minor variations in peak current of fabricated AgCuO NPs/fCNTs/SPCEs occurred. Further, the RSD of peak currents obtained for all the 10 modified electrodes was evaluated and was found as 3.01%, which is very low. Hence, it is obvious that the proposed APAP sensor is evidence of outstanding reproducibility.

#### 3.2.5. Detection of APAP in Real Samples

Practical feasibility of proposed APAP sensor based on AgCuO NPs/fCNTs was analyzed. In this experiment, acetaminophen tablet and human urine were considered as real samples, and the DPV responses of the proposed sensor in these real samples are noted. Table 2 shows the results obtained from this experiment conducted using the standard addition method. The recovery values and RSD of peak currents were calculated from the voltametric responses and are provided in Table 2. The proposed APAP sensor exhibited excellent recovery values for all the real samples in the range of 96.86% to 102.01% and showcased very low RSD values. The maximum RSD value observed in this experiment is merely 3.67%. Therefore, by conducting this investigation, excellent practical feasibility of APAP sensor was confirmed.

## 4. Conclusions

A novel AgCuO NPs/fCNTs hybrid nanocomposite was successfully prepared based on the sonochemical technique for effective electrochemical determination of APAP. Sugar apple-like CuO nanoparticles doped with silver were synthesized, and CNTs functionalization with highly reactive amine group was performed. Various properties of the prepared materials were analyzed using significant characterization techniques. The resultant low-detection limit of 4 nM, high sensitivity of 30.45 μA μM^−1^ cm^−2^, and broad linear ranges from 0.02–3.77 and 3.77–90.02 μM exhibited by the APAP sensor proved its excellent efficiency. The proposed sensor showcased significant traits of a good electrochemical sensor, such as selectivity, stability, repeatability, and reproducibility. The practical applicability of the proposed sensor was carried out in acetaminophen tablet and human urine, resulting in excellent recovery larger than 96.86% and a maximum RSD of 3.67%. Based on the inferences, we can conclude the AgCuO NPs/fCNTs hybrid composite-based sensor is excellent in nanomolar determination of APAP.

## Figures and Tables

**Table 1:** Comparative evaluation of AgCuO NPs/fCNTs-based electrode with other modified electrodes toward APAP detection.

Modified Electrode	Detection Technique	LOD (µM)	Linear Range (µM)	pH	References
GCE/Cu^2+^@PDA-MWCNTs	DPV	0.87	5–75	2	[44]
Pt-Co/NPs/3,4,DHPID/CPE	SWV	0.6	1–850	7	[45]
PANI/MWCNTs/GCE	SWV	0.25	1–100	5.5	[46]
CeBiOx NFs/SPE	DPV	0.2	2.5–130	7.4	[47]
AuNPs@TC8A/GN/GCE	DPV	0.1	0.5–120	7	[48]
MIP–MWCNT/GCE	LSV	0.08	0.2–40	7	[49]
RGO-CB-CTS/GCE	SWV	0.053	2.8–19	6.2	[50]
MIP/GO@COF/GCE	DPV	0.032	0.05–20	7	[51]
MWCNTs/CTS–Cu/GCE	DPV	0.024	0.1–200	7	[52]
CuO-Au/MWCNTs/GCE	DPV	0.016	0.2–6.0	7.5	[53]
La^3+^-CuO/MWCNTs/GCE	DPV	0.014	0.5–900	7	[54]
MWCNT-βCD/GCE	DPV	0.011	0.05–1, 1–300	7.4	[55]
MWCNT/ZnO-Au/GCE	SWV	0.009	0.05–20	7	[56]
AgCuO NPs/fCNTs/SPCE	DPV	0.004	0.02–3.77, 3.77–90.02	7	This work

Abbreviations: PDA—polydopamine; 3,4,DHPID—2-(3,4-dihydroxyphenethyl)isoindoline-1,3-dione; CPE—carbon paste electrode; SWV—square wave voltammetry; PANI—polyaniline; CeBiOx—cerium bismuth oxide; NFs—nanofibers; SPE—screen-printed electrode; AuNPs—gold nanoparticles; TC8A—thiolated calix [8] arene; GN—graphene nanosheets; MIP—molecularly imprinted polymer; LSV—linear sweep voltammetry; RGO—reduced graphene oxide; CB—carbon black; CTS—chitosan; NF—nafion; COF—covalent organic framework; La^3+^—lanthanum; β-CD—β-Cyclodextrin; ZnO—zinc oxide.

**Table 2:** Determination of APAP in real samples using AgCuO NPs/fCNTs/SPCE.

Sample	Added (µM)	Found (µM)	Recovery (%)	RSD ^k^
Acetaminophen tablet	5	5.02	100.4	2.34
	10	10.09	102.93	2.04
	20	20.4	102.01	1.6
Human urine	5	4.84	96.86	3.67
	10	9.69	96.93	2.66
	20	19.82	99.11	2.89

^k^: RSD of three measurements.

## Data Availability

Not applicable.

## Supplementary Materials

The following supporting information can be downloaded at: https://www.mdpi.com/article/10.3390/s23010379/s1.

## References

[1] Sodhi M., Etminan M. (2020). Safety of ibuprofen in patients with COVID-19: Causal or confounded?. Chest.

[2] Morley J.E., Vellas B. (2020). COVID-19 and older adult. J. Nutr. Health Aging.

[3] Day M. (2020). COVID-19: Ibuprofen should not be used for managing symptoms, say doctors and scientists. Br. Med. J..

[4] Bhushan B., Apte U. (2019). Liver regeneration after acetaminophen hepatotoxicity: Mechanisms and therapeutic opportunities. Am. J. Pathol..

[5] Black M. (1984). Acetaminophen hepatotoxicity. Annu. Rev. Med..

[6] Larson A.M., Polson J., Fontana R.J., Davern T.J., Lalani E., Hynan L.S., Reisch J.S., Schiødt F.V., Ostapowicz G., Shakil A.O. (2005). Acetaminophen-induced acute liver failure: Results of a United States multicenter, prospective study. Hepatology.

[7] Mofenson H.C., Caraccio T.R., Nawaz H., Steckler G. (1991). Acetaminophen induced pancreatitis. J. Toxicol. Clin. Toxicol..

[8] Davenport A., Finn R. (1988). Paracetamol (acetaminophen) poisoning resulting in acute renal failure without hepatic coma. Nephron.

[9] Shah A.D., Wood D.M., Dargan P.I. (2011). Understanding lactic acidosis in paracetamol (acetaminophen) poisoning. Br. J. Clin. Pharmacol..

[10] Choueiri T.K., Je Y., Cho E. (2014). Analgesic use and the risk of kidney cancer: A meta-analysis of epidemiologic studies. Int. J. Cancer.

[11] Boussetta K., Ponvert C., Karila C., Bourgeois M.L., de Blic J., Scheinmann P. (2005). Hypersensitivity reactions to paracetamol in children: A study of 25 cases. Allergy.

[12] Ferri F.F. (2016). Ferri’s Clinical Advisor 2017 E-Book: 5 Books in 1.

[13] Daly F.F.S., Fountain J.S., Murray L., Graudins A., Buckley N.A. (2008). Guidelines for the management of paracetamol poisoning in Australia and New Zealand-explanation and elaboration. Med. J. Aust..

[14] Hojjati-Najafabadi A., Mansoorianfar M., Liang T., Shahin K., Karimi-Maleh H. (2022). A review on magnetic sensors for monitoring of hazardous pollutants in water resources. Sci. Total Environ..

[15] Karimi-Maleh H., Darabi R., Shabani-Nooshabadi M., Baghayeri M., Karimi F., Rouhi J., Alizadeh M., Karaman O., Vasseghian Y., Karaman C. (2022). Determination of D&C Red 33 and Patent Blue V Azo dyes using an impressive electrochemical sensor based on carbon paste electrode modified with ZIF-8/g-C_3_N_4_/Co and ionic liquid in mouthwash and toothpaste as real samples. Food Chem. Toxicol..

[16] Cheraghi S., Taher M.A., Karimi-Maleh H., Karimi F., Shabani-Nooshabadi M., Alizadeh M., Al-Othman A., Erk N., Raman P.K.Y., Karaman C. (2022). Novel enzymatic graphene oxide based biosensor for the detection of glutathione in biological body fluids. Chemosphere.

[17] Karimi-Maleh H., Beitollahi H., Kumar P.S., Tajik S., Jahani P.M., Karimi F., Karaman C., Vasseghian Y., Baghayeri M., Rouhi J. (2022). Recent advances in carbon nanomaterials-based electrochemical sensors for food azo dyes detection. Food Chem. Toxicol..

[18] Sebastian N., Yu W.-C., Balram D. (2019). Electrochemical detection of an antibiotic drug chloramphenicol based on a graphene oxide/hierarchical zinc oxide nanocomposite. Inorg. Chem. Front..

[19] Balram D., Lian K.-Y., Sebastian N. (2018). A novel electrochemical sensor based on flower shaped zinc oxide nanoparticles for the efficient detection of dopamine. Int. J. Electrochem. Sci..

[20] Buledi J.A., Mahar N., Mallah A., Solangi A.R., Palabiyik I.M., Qambrani N., Karimi F., Vasseghian Y., Karimi-Maleh H. (2022). Electrochemical quantification of mancozeb through tungsten oxide/reduced graphene oxide nanocomposite: A potential method for environmental remediation. Food Chem. Toxicol..

[21] Wei C., Zou X., Liu Q., Li S., Kang C., Xiang W. (2020). A highly sensitive non-enzymatic glucose sensor based on CuS nanosheets modified Cu2O/CuO nanowire arrays. Electrochim. Acta.

[22] Yang L., Chu D., Wang L. (2015). Porous hexapod CuO nanostructures: Precursor-mediated fabrication, characterization, and visible-light induced photocatalytic degradation of phenol. Mater. Lett..

[23] Zhang J., Liu J., Peng Q., Wang X., Li Y. (2006). Nearly monodisperse Cu_2_O and CuO nanospheres: Preparation and applications for sensitive gas sensors. Chem. Mater..

[24] Yuan W., Qiu Z., Chen Y., Zhao B., Liu M., Tang Y. (2018). A binder-free composite anode composed of CuO nanosheets and multi-wall carbon nanotubes for high-performance lithium-ion batteries. Electrochim. Acta.

[25] Luo L., Zhu L., Wang Z. (2012). Nonenzymatic amperometric determination of glucose by CuO nanocubes–graphene nanocomposite modified electrode. Bioelectrochemistry.

[26] John S., Vadla S.S., Roy S.C. (2019). High photoelectrochemical activity of CuO nanoflakes grown on Cu foil. Electrochim. Acta.

[27] Zhang Z., Zada A., Cui N., Liu N., Liu M., Yang Y., Jiang D., Jiang J., Liu S. (2021). Synthesis of ag loaded ZnO/BiOCl with high photocatalytic performance for the removal of antibiotic pollutants. Crystals.

[28] Li Y., Wan M., Yan G., Qiu P., Wang X. (2021). A dual-signal sensor for the analysis of parathion-methyl using silver nanoparticles modified with graphitic carbon nitride. J. Pharm. Anal..

[29] Sebastian N., Yu W.-C., Balram D. (2022). Ultrasensitive Electrochemical Detection and Plasmon-Enhanced Photocatalytic Degradation of Rhodamine B Based on Dual-Functional, 3D, Hierarchical Ag/ZnO Nanoflowers. Sensors.

[30] Sebastian N., Yu W.-C., Balram D., Noman M.T., Amor N. (2022). Silver doped dodecahedral metal-organic framework anchored RGO nanosheets for nanomolar quantification of priority toxic pollutant in aquatic environment. J. Alloys Compd..

[31] Inam A.K.M.S., Angeli M.A.C., Shkodra B., Douaki A., Avancini E., Magagnin L., Lugli P. (2022). Flexible screen-printed amperometric sensors functionalized with spray-coated carbon nanotubes and electrodeposited Cu nanoclusters for nitrate detection. IEEE Sens. J..

[32] Boumya W., Taoufik N., Achak M., Barka N. (2021). Chemically modified carbon-based electrodes for the determination of paracetamol in drugs and biological samples. J. Pharm. Anal..

[33] Mallakpour S., Khadem E. (2016). Carbon nanotube–metal oxide nanocomposites: Fabrication, properties and applications. Chem. Eng. J..

[34] Flahaut E., Peigney A., Laurent C., Marliere C., Chastel F., Rousset A. (2000). Carbon nanotube–metal–oxide nanocomposites: Microstructure, electrical conductivity and mechanical properties. Acta Mater..

[35] Sebastian N., Yu W.-C., Balram D. (2020). Synthesis of amine-functionalized multi-walled carbon nanotube/3D rose flower-like zinc oxide nanocomposite for sensitive electrochemical detection of flavonoid morin. Anal. Chim. Acta.

[36] Sebastian N., Yu W.-C., Hu Y.-C., Balram D., Yu Y.-H. (2019). Sonochemical synthesis of iron-graphene oxide/honeycomb-like ZnO ternary nanohybrids for sensitive electrochemical detection of antipsychotic drug chlorpromazine. Ultrason. Sonochem..

[37] Patterson A.L. (1939). The Scherrer formula for X-ray particle size determination. Phys. Rev..

[38] Balzar D., Ledbetter H. (1993). Voigt-function modeling in Fourier analysis of size-and strain-broadened X-ray diffraction peaks. J. Appl. Crystallogr..

[39] Tran T.H., Nguyen V.T. (2014). Copper oxide nanomaterials prepared by solution methods, some properties, and potential applications: A brief review. Int. Sch. Res. Not..

[40] Amirian M., Chakoli A.N., Cai W., Sui J. (2013). Effect of functionalized multiwalled carbon nanotubes on thermal stability of poly (L-LACTIDE) biodegradable polymer. Sci. Iran..

[41] Kaur A., Mann S., Goyal B., Pal B., Goyal D. (2016). CuO nanostructures of variable shapes as an efficient catalyst for [3 + 2] cycloaddition of azides with terminal alkyne. RSC Adv..

[42] Miner D.J., Rice J.R., Riggin R.M., Kissinger P.T. (1981). Voltammetry of acetaminophen and its metabolites. Anal. Chem..

[43] Beitollahi H., Ardakani M.M., Ganjipour B., Naeimi H. (2008). Novel 2, 2′-[1,2-ethanediylbis (nitriloethylidyne)]-bis-hydroquinone double-wall carbon nanotube paste electrode for simultaneous determination of epinephrine, uric acid and folic acid. Biosens. Bioelectron..

[44] Shahbakhsh M., Noroozifar M. (2018). Copper polydopamine complex/multiwalled carbon nanotubes as novel modifier for simultaneous electrochemical determination of ascorbic acid, dopamine, acetaminophen, nitrite and xanthine. J. Solid State Electrochem..

[45] Karimi-Maleh H., Hatami M., Moradi R., Khalilzadeh M.A., Amiri S., Sadeghifar H. (2016). Synergic effect of Pt-Co nanoparticles and a dopamine derivative in a nanostructured electrochemical sensor for simultaneous determination of N-acetylcysteine, paracetamol and folic acid. Microchim. Acta.

[46] Li M., Jing L. (2007). Electrochemical behavior of acetaminophen and its detection on the PANI–MWCNTs composite modified electrode. Electrochim. Acta.

[47] Cao F., Dong Q., Li C., Chen J., Ma X., Huang Y., Song D., Ji C., Lei Y. (2018). Electrochemical sensor for detecting pain reliever/fever reducer drug acetaminophen based on electrospun CeBiOx nanofibers modified screen-printed electrode. Sens. Actuators B Chem..

[48] Liu W., Shi Q., Zheng G., Zhou J., Chen M. (2019). Electrocatalytic oxidation toward dopamine and acetaminophen based on AuNPs@ TCnA/GN modified glassy carbon electrode. Anal. Chim. Acta.

[49] Peng Y., Wu Z., Liu Z. (2014). An electrochemical sensor for paracetamol based on an electropolymerized molecularly imprinted o-phenylenediamine film on a multi-walled carbon nanotube modified glassy carbon electrode. Anal. Methods.

[50] Baccarin M., Santos F.A., Vicentini F.C., Zucolotto V., Janegitz B.C., Fatibello-Filho O. (2017). Electrochemical sensor based on reduced graphene oxide/carbon black/chitosan composite for the simultaneous determination of dopamine and paracetamol concentrations in urine samples. J. Electroanal. Chem..

[51] Sun Y., He J., Waterhouse G.I.N., Xu L., Zhang H., Qiao X., Xu Z. (2019). A selective molecularly imprinted electrochemical sensor with GO@ COF signal amplification for the simultaneous determination of sulfadiazine and acetaminophen. Sens. Actuators B Chem..

[52] Mao A., Li H., Jin D., Yu L., Hu X. (2015). Fabrication of electrochemical sensor for paracetamol based on multi-walled carbon nanotubes and chitosan–copper complex by self-assembly technique. Talanta.

[53] Shaikshavali P., Reddy T.M., Palakollu V.N., Karpoormath R., Rao Y.S., Venkataprasad G., Gopal T.V., Gopal P. (2019). Multi walled carbon nanotubes supported CuO-Au hybrid nanocomposite for the effective application towards the electrochemical determination of acetaminophen and 4-aminophenol. Synth. Met..

[54] Foroughi M.M., Jahani S., Nadiki H.H. (2019). Lanthanum doped fern-like CuO nanoleaves/MWCNTs modified glassy carbon electrode for simultaneous determination of tramadol and acetaminophen. Sens. Actuators B Chem..

[55] Alam A.U., Qin Y., Howlader M.M.R., Hu N.-X., Deen M.J. (2018). Electrochemical sensing of acetaminophen using multi-walled carbon nanotube and β-cyclodextrin. Sens. Actuators B Chem..

[56] Kenarkob M., Pourghobadi Z. (2019). Electrochemical sensor for acetaminophen based on a glassy carbon electrode modified with ZnO/Au nanoparticles on functionalized multi-walled carbon nano-tubes. Microchem. J..

