# Supplementary material for: Electrocatalytic Platform Based on Silver-Doped Sugar Apple-like Cupric Oxide Embedded Functionalized Carbon Nanotubes for Nanomolar Detection of Acetaminophen (APAP)
Source: Sensors (Basel). 2022 Dec 29;23(1):379. doi: 10.3390/s23010379 (PMC9823579; doi:10.3390/s23010379)
Supplement: Supplementary file 1 [file sensors-23-00379-s001.zip › sensors-2081758-supplementary.pdf]

Electrocatalytic Platform Based on Silver-Doped Sugar Apple-like Cupric Oxide Embedded Functionalized Carbon Nanotubes for Nanomolar Detection of Acetaminophen (APAP)

Deepak Balram [1], Kuang-Yow Lian [1,*] and Neethu Sebastian [2]

[1] Department of Electrical Engineering, National Taipei University of Technology, No. 1, Section 3, Zhongxiao East Road, Taipei 106, Taiwan
[2] Institute of Organic and Polymeric Materials, Department of Molecular Science and Engineering, National Taipei University of Technology, No. 1, Section 3, Zhongxiao East Road, Taipei 106, Taiwan
* Correspondence: kylian@ntut.edu.tw

Determination and evaluation of electroactive surface area

Electroactive surface area has a crucial role in determining the electrocatalytic performance of a material. Keeping this fact in mind, the electroactive surface area of AgCuO NPs/fCNTs was evaluated. Cyclic voltammetry technique was used for this evaluation by recoding the resultant voltammetric curves of AgCuO NPs/fCNTs/SPCE and bare SPCE kept in 0.1 M KCl comprising 5 mM $[Fe(CN)_6]^{-3/-4}$ at different scan rates (0.02 – 0.2 Vs^{-1}). The resultant voltammetric curves for unmodified electrode and AgCuO NPs/fCNTs/SPCE are depicted in Figures 6C and 6E respectively. In order to determine the electroactive surface area, we need to evaluate the slope of resultant voltammetric curves. Hence, we have determined the regression equations based on the linear plots between square root of scan rate and redox peak currents of bare SPCE and AgCuO NPs/fCNTs/SPCE. Fig. 6D and 6F represents the aforementioned linear plots of bare SPCE and AgCuO NPs/fCNTs/SPCE determined from the corresponding voltammetric response. From these linear plots, we have determined respective slope and used this value in the Randles Sevcik equation for determining electroactive surface area of both bare SPCE and AgCuO NPs/fCNTs/SPCE.

$$I_p = (2.69 \times 10^5)\, A D^{1/2} v^{1/2} n^{3/2} C \qquad (1)$$

where I_p is the oxidation peak current, A represents active surface area, D denotes diffusion coefficient, C is the concentration, n denotes electrons, and v is the scan rate. The resultant electroactive surface area of bare SPCE and AgCuO NPs/fCNTs/SPCE we

have determined based on Randles Sevcik equation are 0.01 cm^2 and 0.26 cm^2 respectively. This result indicates that the electroactive surface area of the developed AgCuO NPs/fCNTs/SPCE is around 18 times of unmodified electrode. As mentioned earlier, higher electroactive surface area of a material results in its superior electrocatalytic activity. Therefore, it is evident that AgCuO NPs/fCNTs/SPCE will exhibit enhanced electrocatalytic performance. This once again confirms that the prepared AgCuO NPs/fCNTs nanocomposite is suitable for development of electrochemical sensor.
